# Geology and Genesis

**Published:** 1862-12

**Authors:** 


HALL'S JOURNAL OF HEALTH.

Vol. IX.] DECEMBER, 1862. [No. 12.

### GEOLOGY AND GENESIS.

A GREAT and an injurious mistake is made by some Bible expositors, in endeavoring to reconcile Bible statements with the claims of science. Very few scientific truths are demonstrable, out of the line of mathematics. Many things appear to be true—seem to be reasonable; but the attempt to square a Bible assertion with a seeming fact, with a "reasonable" assumption, is a folly and a sin. If any fact, or truth, or proposition, is an absolute demonstration, founded on no bare assumption, and such fact, or truth, or proposition, is in direct and incontrovertible opposition to a Bible statement, then it may be time to "inquire into it;" but sooner than that, is not wise.

There is no fact in Geology more universally admitted, than that an uncounted multitude of years were required for arranging the present order of things, as to the surface of the earth; that ages must have been necessary to form its various layers or strata; and since the Bible speaks of "days," as to the time consumed, the conclusion is being very generally adopted, that the "day" of the first chapter of Genesis meant an indefinite duration of time. Hugh Miller, the Napoleon of modern geology, as to its mere physical observed facts, favors this interpretation, which derives an influence from his name, altogether beyond its merits; because one of his earliest fancies was that of a fanatic, or lunarian, in reference to some vision or "apparition." His last act was that of a madman; while, between the two, there were indications of mental weakness, which were nothing short of foolish fancies. Such a man's testimony, such a man's opinions, such a man's theories, should be received only with great deliberation, with considerable hesitation, when they bear on religious truth; and are absolutely worth nothing beyond the corroboration which whole facts afford.

The dogmatism of the dolt will not be fallen into here. It is preferred to leave the subject open, only recording a caveat against committing the interpretation, as to the meaning of any portion of the Bible, to any man, even though it be in connection with a subject of which he was a perfect master.

The power which commanded, "Let there be light, and light was," is adequate to the making of this world, and a million more like it, in six days. That Power is above law, and can suspend law by the fiat of his mouth; and by the same, can make it. All physical law is nothing more or less than the will of God. If he chose to make this world in a week, or minute, or second, by any agency, that agency is law enough.

We will not commit ourselves by saying that the first chapter of Genesis means this, that, or the other; nor will we aver that it does not or cannot mean anything that may be affirmed; we only want that it shall not be committed, by common consent, to any human interpretation, simply because we cannot tell what developments of natural truth are to be made in the course of events.

One principle of interpretation, which ought never to be lost sight of, is simply this: any word or sign, any letter or hieroglyphic in a document, ought to have the same meaning attached to it throughout. If a day of work means an indefinite duration, so does a day of rest; and to rest indefinitely, whether for Maker or man, is an absurdity. For man to work an indefinite time, is a physical impossibility. If a day of work means twenty-four hours, so does a day of rest. If a day of work means a million of years, so does a day of rest. Hence, we prefer to accept the interpretation which the record makes of itself, that a day consisted of an evening, running back to the morning which preceded it, and including both morning and evening in that "day one," which is the Hebrew form of expression.

The "beginning" of the first verse may have been ages before the commencement of the description; and this earth may have existed for ages more, as a wild waste of waters, in shapelessness and darkness. These propositions are neither affirmed nor denied; our great idea is non-committal; but that, after the second verse, there is anything inconsistent with a literal interpretation of the record, we cannot see.

Geologists are firm in their conviction, that there was a duration of ages before even the lowest forms of vegetation existed; that then, in the lapse of other vast cycles of time, creeping things and reptiles came; and next, animals; and, last of all, man. Geology shows, beyond a peradventure, that such was the order of their coming; and this glorious first chapter of Genesis, the oldest and grandest written record on the globe, says the same thing. But when Geology asserts, that it must have required countless ages to have brought about these changes and progressions, it is merely an assumption. The Bible says "a day;" Geology says that day was one of years, centuries, cycles. How long that day was, the Bible does not say; Geology speaks for it, defines it. But Geology wasn't there, hence can know nothing about it. And a geological "must," which calls in question a Bible statement, which requires the modification of a Bible record, is an impudent presumption.

If, then, the unlearned Christian has to meet the pretentious lore, and the "great swelling words" of infidel geologists, as to the meaning of the Bible account of creation, it is sufficient to answer, "The Sacred Volume says nothing as to the age of the earth before light, and dry land, and sea, and vegetation, and insect, and animal, and man, came. But when they did come, it says they came in a 'day'—that that day included an evening and a morning; whether it was a day of twenty-four hours, or of twenty-four years, or millions of years, it does not say; it was not necessary for it to have been said. Here I leave it. If you assert that the 'day' meant long cycles of time, prove it by witnesses on the spot; and when you have done so, you will have shown nothing contrary to the Bible record."

